# Topoisomerase Inhibitors Increase Episomal DNA Expression by Inducing the Integration of Episomal DNA in Hepatic Cells

**DOI:** 10.3390/pharmaceutics15102459

**Published:** 2023-10-13

**Authors:** Andoni Gómez-Moreno, Enara San Sebastian, Jennifer Moya, Pilar Gomollón-Zueco, Sergio Isola, África Vales, Gloria González-Aseguinolaza, Carmen Unzu, Urtzi Garaigorta

**Affiliations:** 1 Departamento de Biología Molecular y Celular, Centro Nacional de Biotecnología-Consejo Superior de Investigaciones Científicas (CNB-CSIC), Calle Darwin 3, 28049 Madrid, Spain; esansebastian@cnb.csic.es (E.S.S.); jmoya@cnb.csic.es (J.M.); pilargomollon@unizar.es (P.G.-Z.); 2 DNA & RNA Medicine Division, CIMA, Universidad de Navarra, Avda Pio XII, 55, 31008 Pamplona, Spain; sisola@unav.es (S.I.); avales@unav.es (Á.V.); ggasegui@unav.es (G.G.-A.); cunzu@unav.es (C.U.)

**Keywords:** topoisomerase inhibitor, camptothecin, etoposide, ssAAV, scAAV, IDLV, ICLV, integration: episomal DNA, gene therapy

## Abstract

Gene therapy is a promising strategy to treat and cure most inherited metabolic liver disorders. Viral vectors such as those based on adeno-associated viruses (AAVs) and lentiviruses (LVs) are used as vehicles to deliver functional genes to affected hepatocytes. Adverse events associated with the use of high vector doses have motivated the use of small molecules as adjuvants to reduce the dose. In this study, we showed that a one-hour treatment with topoisomerase inhibitors (camptothecin and etoposide) prior to viral transduction is enough to increase AAV and LV reporter expression in non-dividing hepatic cells in culture. Topoisomerase inhibitors increased both integration-competent (ICLV) and integration-deficient (IDLV) LV-derived expression, with a much stronger increase in the IDLV transduction system. In agreement with that, topoisomerase inhibitors increased viral genome integration in both strains, with a greater impact on the IDLV strain, supporting the idea that topoisomerase inhibitors increased episomal DNA integration, especially when viral integrase activity is abolished. These effects correlated with an increase in the DNA damage response produced by the treatments. Our study highlights the need to monitor DNA damage and undesired integration of viral episomal DNAs into the host genome when studying chemical compounds that increase viral transduction.

## 1. Introduction

The liver is an essential organ involved in a variety of functions including detoxification, vitamin and glucose storage, iron metabolism, regulation of hormones, production of most plasma proteins, and metabolism of carbohydrates, fat and proteins [1]. Hepatocytes constitute the bulk of cells in the liver parenchyma and are affected by the majority of monogenic liver inherited disorders. Consequently, the liver is a prime target for gene therapy, and many liver-targeted therapies are under investigation [2,3,4,5,6,7,8,9]. A common gene therapy strategy consists of gene supplementation, where a functional form of an affected gene is delivered to the target cells, which allows the phenotypic correction of a disorder. For decades, adeno-associated viruses (AAVs) and lentiviruses (LVs) have been used as vectors for gene supplementation [10,11]. These vectors are able to release ectopic DNA in the nucleus of transduced hepatocytes that is responsible for transgene expression.

AAVs have a single-stranded DNA (ssDNA) genome of 4.7 kb length that when they reach the nucleus are converted into double-stranded DNA (dsDNA) by host proteins prior to transcription [12]. For gene therapy applications, recombinant AAVs (rAAV) are generated, where the ssDNA viral genome is replaced by a therapeutic expression cassette flanked by the AAV inverted terminal repeats (ITR). Instead, self-complementary AAV vectors (scAAV) were designed to deliver their genome in a dsDNA form due to a truncation in one of the ITRs [12,13]. Transduction with scAAV vectors produces higher levels of the transgene and in a faster way compared to single-stranded AAV (ssAAV) vectors [14]. However, its cloning capacity is considerably reduced [12]. rAAV episomal DNA does not replicate and it can be silenced or lost, especially during cell division [11]. Nevertheless, in non-growing tissues, rAAV gene expression can last for years [15]. Integration of rAAV genomes is rare, random and occurs preferentially at chromosomal breakage sites [16]. Integrated rAAV genome expression might be silenced through histone modification and chromatin condensation [17,18,19]. Thus, rAAV transgene expression is primarily derived from episomal DNA forms. 

LVs, as well as recombinant LV vectors (rLVs), used in gene therapy applications, have a single-stranded positive polarity RNA genome that is retrotranscribed into dsDNA by the viral retrotranscriptase [10], generating the DNA template for integration. Integration into the host cell is driven by the viral integrase and it is required for viral gene expression [20,21,22]. The cloning capacity of rLV vectors exceeds that of rAAV vectors, reaching up to 10 kb in size [23]. Integration overcomes the loss of episomal DNA during cell division; however, concerns about insertional mutagenesis and oncogenic potential exist. To avoid these problems, integration deficient lentiviral vectors (IDLV) were developed. IDLVs carry point mutations in the viral integrase that avoid viral genome integration [24]. As a consequence, non-integrated viral genomes accumulate in the form of 1-long terminal repeat (LTR) and 2-LTR circular episomal DNA [25,26], which maintain transgene expression capacity, albeit to a reduced level compared to their integration-competent LV (ICLV) counterparts [27]. As it occurs with AAV expression, IDLV episomal DNA expression can be lost upon cell division [28,29].

In the last few decades, rAAV and rLV vectors have been extensively studied in preclinical settings as viral gene therapy vehicles. In order to maintain efficient therapeutic protein expression, high vector doses are sometimes required, which can induce activation of innate and adaptive responses against the viral vectors decreasing its transgene expression [11]. Moreover, it can also induce adverse effects, including hepatotoxicity, nephrotoxicity, neurotoxicity and viral genome integration into the host genome, causing undesired mutations and driving proto-oncogene expression [30,31]. For these reasons, development of more efficient and safer viral vectors, as well as methods to improve viral vector transduction, are still needed. In this regard, several authors have explored the possibility of using chemical compounds as adjuvants of viral vector transduction. Indeed, DNA-damaging agents, including topoisomerase I (TOP1) and II (TOP2) inhibitors (e.g., camptothecin (CPT) and Etoposide (Eto), respectively), produced an increase in the rAAV transduction efficiency in cell culture in vitro [32,33]. Topoisomerase inhibitors are known to induce double-strand break formation in cellular DNA [34]. In this context, DNA damage breakage sites induced by Eto and γ-irradiation increased rAAV genome integration [16], suggesting that the increased rAAV transduction observed by other authors might be a consequence of AAV DNA integration induced by the treatments. However, epigenetic mechanisms have been shown to trigger silencing of rAAV genome expression from integrated viral DNA [17,18,19]. Thus, it is still unclear the mechanism by which topoisomerase inhibitors increase AAV-derived transgene expression. 

Integration is a key process in ICLV infection that is driven by viral integrase and allows robust lentivirus gene expression [20,21,22]. It has been reported that integration-defective human immunodeficiency virus (HIV) strains are still capable of integrating their genomes, albeit at very low levels compared to their ICLV counterparts [35,36]. Compounds that increase ICLV transduction have also been identified [37]. Groschel et al. described that CPT and Eto induce cell cycle arrest and promote ICLV integration into the host chromosomal DNA [38]. Induction of DNA damage by treatment with H_2_O_2_ or by exposure to γ-irradiation, prior to transduction, increased IDLV integration [39]. Moreover, the presence of DNA damage agents, such as bleomycin and Eto, during viral transduction was able to increase IDLV but not ICLV viral genome copy numbers in macrophages [35]. In contrast to those observations, a different study, performed in monocyte-derived macrophages, showed that induction of DNA damage by CPT and Eto blocks HIV-1 infection after completion of viral DNA synthesis, at the step involving 2-LTR circle and provirus formation [40]. 

Nevertheless, how topoisomerase inhibitors modulate viral vector transgene expression remains unclear and further research is needed to shed light on the specific mechanisms responsible for the observed effects. In this study, we use a systematic approach to evaluate the phenotypic effect of CPT and Eto treatments on rAAV and rLV vector transduction in non-dividing hepatic cells in vitro. On the one hand, we analyzed the effect of the compounds on the induction of DNA damage and cell viability and correlated the results with a quantitative analysis of viral vector gene expression. On the other hand, we used different rAAV (ssAAV vs. scAAV) and rLV (ICLV vs. IDLV) vectors, all expressing the same mCherry reporter gene, to show that CPT and Eto increase their expression by increasing the integration of viral episomal genomes in hepatic cells. 

## 2. Materials and Methods

### 2.1. Cells, Antibodies, Plasmids and Reagents

The origins of HepG2-NTCP [41] and HEK-293T [42] cells have been described previously. All cells were maintained in Dulbecco’s modified Eagle’s medium (DMEM) (cat. nº: D6429-500ML, Sigma-Aldrich, St. Louis, MI, USA) supplemented with 10% fetal bovine serum (FBS; cat. nº: 35-079-CF, Corning, NY, USA), 10 mM HEPES (cat. nº: H0887-100ML, Sigma-Aldrich), 100 units/mL penicillin and 100 mg/mL streptomycin, (cat. nº: P4333-100ML, Sigma-Aldrich) and 2 mM L-glutamine (cat. nº: G7513-100ML, Sigma-Aldrich) in 5% CO_2_ at 37 °C. To avoid cell overgrowth during the experiments, culture medium was supplemented with 2% dimethyl sulfoxide (DMSO; cat. nº: D2650, Sigma-Aldrich) starting one day before any treatment was applied or before any viral transduction and it was kept during the entire length of the experiments. Camptothecin (CPT; cat. nº: S1288, Selleckchem, Houston, TX, USA), etoposide (Eto; cat. nº: S1225, Selleckchem) and actinomycin D (ActD; cat. nº: A9415, Sigma-Aldrich) were obtained from commercial sources. Aliquots of stock solutions were prepared in DMSO and stored frozen at −20 °C until they were used in the experiments at the concentrations indicated in each experiment. The pLenti-C-Myc-DDK plasmid (cat. nº: PS100064, Origene, Rockville, MD, USA) was used for the cloning of the mCherry reporter gene. The lentivirus packaging vectors (pRSV-Rev, pMDLg-pRRE and pMD2.G) were obtained from the Addgene repository (Addgene plasmid #12251, #12253 and #12259). The AAV packaging plasmid pDP3 was obtained from PlasmidFactory (#PF0433, Germany).

### 2.2. Molecular Cloning

#### 2.2.1. Generation of pLenti-mCherry-NLS Plasmid for Lentiviral Production

The DNA sequence corresponding to the mCherry open reading frame was amplified by PCR using the following primers: AsiSI-mCherry-Fwd (5′-AGCTGCGATCGCATGGTGAGCAAGGGCGAGG-3′) and MluI-mCherry-Rev (5′-AGCTACGCGTCTTGTACAGCTCGTCCATGCC-3′), and the CHC-mCherry plasmid as a template with the Supreme NZYProof DNA polymerase (cat. nº: MB28302, NzyTech, Lisbon, Portugal), according to the manufacturer´s instructions. The polymerase chain reaction (PCR) conditions were as follows: 1 cycle at 96 °C for 4 min; 25 cycles of 30 s at 96 °C, 30 s at 60 °C and 30 s at 72 °C; and 1 cycle at 72 °C for 5 min. The column-purified PCR product and the destination plasmid vector pLenti-C-Myc-DDK were subjected to AsiSI (cat. nº: R0630L, New England Biolabs (NEB), Ipswich, MA, USA) and MluI-HF (cat. nº: R3198L, NEB) digestion and subsequently ligated using T4 DNA ligase (cat. nº: M0202S, NEB). The sequence of the resulting pLenti-mCherry plasmid was verified by Sanger sequencing.

The pLenti-mCherry-NLS plasmid was generated by the insertion of the nuclear localization signal (NLS) of the cellular c-myc gene followed by three consecutive stop codons at the end of the mCherry open reading frame in the parental pLenti-mCherry plasmid. To do so, the following oligonucleotides 5′-CGCGTCCTGCTGCTAAGAGAGTGAAACTGGATTGATAATAGC-3′ and 5′-TCGAGCTATTATCAATCCAGTTTCACTCTCTTAGCAGCAGGA-3′ were subjected to annealing reactions to produce a short double-stranded DNA product with 5′ and 3′ overhang ends ready for directed ligation into a MluI-HF and XhoI (cat. nº: R0146L, NEB) digested pLenti-mCherry plasmid. The sequence of the resulting pLenti-mCherry-NLS plasmid was verified by Sanger sequencing and was used for the generation of lentiviruses as described below.

#### 2.2.2. Generation of pAAV-EALBAAT-mCherry-NLS and dsAAV-EALBAAT-mCherry-NLS Plasmids for AAV Production

The DNA sequence corresponding to the mCherry open reading frame followed by the NLS sequence of c-myc was amplified by PCR from the pLenti-mCherry-NLS plasmid using the following primers: 5′-ACTGCCATGGTGAGCAAGGGCGAGGAGG-3′ and 5′-ACTGGAATTCTAGAGTCGCGGCCGCTATTATCAATCCAGTTTCACTCTC-3′, and the Speedy NZYTaq 2× Green Master Mix (cat. nº: MB36201, NzyTech) following the manufacturer’s instructions. The column-purified PCR product and the destination plasmid vector pAAV-EALBAAT-EGFP-PA were subjected to NcoI-HF (cat. nº: R3193L, NEB) and EcoRI-HF (cat. nº: R3101L, NEB) digestion and subsequently ligated using T4 DNA ligase. The expression cassette was then cloned in a backbone plasmid with a mutated ITR to generate the self-complementary rAAV (dsAAV). The sequence of the resulting pAAV and dsAAV-EALBAAT-mCherry-NLS plasmids was verified by Sanger sequencing. 

#### 2.2.3. Generation of pMDLg_pRRE-D64A Plasmid for Integration-Deficient Lentivirus (IDLV) Production

The D64A mutation [43] was introduced by site-directed mutagenesis in the parental pMDLg_pRRE lentivirus packaging plasmid. To do so, a mutagenic PCR was performed using 5′-ATGGCAGCTAGCTTGTACACATTTAG-3′ and 5′-ATTCCTGGGCTACAGTCTAC-3′ primers with Supreme NZYProof DNA polymerase (cat. nº: MB28301, NzyTech) and the PCR product was subsequently circularized with KLD reaction mix (cat. nº. M0554S, NEB) following the manufacturer’s instructions. The sequence of the resulting pMDLg_pRRE-D64A plasmid was verified by Sanger sequencing and was used for the generation of IDLVs as described below.

### 2.3. Viral Vector Production and Titration

#### 2.3.1. ICLV and IDLV Production and Titration

ICLV and IDLV expressing the mCherry-NLS reporter gene in their genome were produced in HEK-293T cells by co-transfection of all necessary packaging plasmids (pRSV-Rev, pMDLg-pRRE and pMD2.G) as previously described [44]. Cell supernatants were collected at 40–48 h post-transfection, filtered through 0.45 µm filters, aliquoted and kept at −80 °C until needed. Titration was carried out by inoculation of HepG2-NTCP cells with two-fold serial dilutions of lentiviral particles. Quantitation of the number of and the mean fluorescence intensity signal in mCherry-positive cells was used to determine the amount of each lentiviral particle to be used in the experiments. As expected, ICLV transduction was much more efficient than IDLV transduction.

#### 2.3.2. rAAV Production and Titration

Production of the rAAVs was performed by double transfection in HEK-293T cells using the pAAV plasmid with the expression cassette and a pDP3 plasmid that includes the *rep* and *cap* AAV genes and the adenovirus genes essential for replication. HEK-293T cells (2% FBS DMEM; 65% cell confluency) were co-transfected using linear polyethyleneimine at 25 kDa (cat. nº: 23966-100, Polysciences, Warrington, PA, USA). Vector particles were obtained from cells and the supernatant. After 72 h, the supernatant was treated with polyethylene glycol solution (PEG8000, 8% final concentration; cat. nº: 89510, Sigma-Aldrich) at 4 °C for 48 h, then centrifuged for 15 min at 3000 rpm, and the viral particles present in the pellet were resuspended in lysis buffer (50 mM Tris-Cl, 150 mM NaCl, 2 mM MgCl_2_, 0.1% Triton X-100) and kept at −80 °C. Cells were treated with lysis buffer and kept at −80 °C. Then, both the supernatant and cells were subjected to 3 freeze/thaw cycles, centrifuged and finally, they were treated with DNase and RNase solutions. This lysate was purified in an iodixanol gradient (15, 25, 40 and 54% iodixanol) by ultracentrifugation (69,000 rpm, 16 °C, 2.5 h; in Beckman type 70 Ti rotor in Beckman) according to previously published methods [45] and concentrated by Ultra-15 mL Amicon columns (cat. nº: C7715, Amicon^®^; Millipore, Bedford, MA, USA). AAV vector genomes were extracted using the High Pure Viral Nucleic Acid Kit (cat. nº: 11858874001, Roche, Switzerland) according to the manufacturer’s specifications. The quality of the vector production was checked by two techniques: viral genome quantification by real-time-quantitative PCR (qPCR) assay and capsid protein detection by sodium dodecyl sulphate–polyacrylamide gel electrophoresis (SDS-PAGE) combined with SYPRO Ruby staining. Real-time-qPCR was performed to quantify the number of AAV genomes (viral genomes (vg)/mL)), using the GoTaq^®^ qPCR Master Mix (cat. nº: A6001, Promega, Madison, WI, USA) and primers specific for ITRs [46], while SDS-PAGE was used to analyze the capsid protein ratio. Vectors were stored at −80 °C until use. 

### 2.4. Compound Treatment

HepG2-NTCP cells were seeded at high density in 12-well plate (4 × 10^5^ cells per well) or 96-well plate (4 × 10^4^ cells per well) formats, as indicated in each experiment. The following day, the medium was discarded and freshly prepared 2% DMSO-containing medium was added to the cells and they were further incubated for another twenty-four hours. The next day, cells were treated for one hour with the indicated concentration of compounds diluted in 2% DMSO-containing medium. After the treatment, the medium was discarded, and cells were processed immediately for double-strand break detection or cytotoxicity analysis, or they were subjected to viral transduction or metabolic labeling as indicated below.

### 2.5. Detection of Double-Strand Breaks by γH2AX Immunofluorescence

HepG2-NTCP cells plated in a 96-well plate format and treated as indicated in each figure legend were subjected to immunofluorescence analysis with specific antibodies to detect the phosphorylated form of H2AX (γH2AX), a marker of double-strand break formation. To do so, the compound-containing medium was discarded right after one hour of treatment. Cells were washed with phosphate-buffered saline (PBS) and incubated in 4% formaldehyde (FA)-PBS solution for twenty minutes at room temperature. Then, they were extensively washed with PBS to remove the fixative and they were processed for immunofluorescence analysis as previously described [47]. In brief, cells were incubated in blocking buffer (1xPBS, 10% FBS, 3% bovine serum albumin (BSA), 0.3% Triton X-100) for one hour at room temperature, washed three times with PBS and then incubated for one hour with a 1:200 dilution of a rabbit monoclonal antibody against γH2AX (cat. nº: 9718, Cell Signaling Technology, Danvers, MA, USA) prepared in binding buffer (1xPBS, 3% BSA, 0.3% Triton X-100). After extensive washes with PBS, cells were incubated for one hour in a mixture of 2 μg/mL Alexa Fluor^TM^ 488-conjugated goat anti-rabbit IgG cross-adsorbed secondary antibodies (cat. nº: A-11008, Invitrogen, Waltham, MA, USA) and 0.5 μg/mL Hoechst 33342 dye (cat. nº: H3570, Invitrogen) for nuclei staining. The fluorescence signal was then imaged and pictures were taken in a SparkCyto plate reader (Tecan, Austria) after extensive washes with PBS. Images were analyzed as indicated below.

### 2.6. Cytotoxicity Analysis

The toxicity of compounds was evaluated by quantitation of metabolic activity using an MTT-formazan assay. HepG2-NTCP cells plated in a 96-well plate format were subjected to compound treatment at concentrations indicated in the corresponding figure legend. One hour later, the culture medium was discarded, cells were washed with complete medium and subjected to MTT assays (cat nº: M2128, Sigma-Aldrich) using previously described procedures [48].

### 2.7. Viral Transduction

Appropriate amounts of lentiviral (ICLV or IDLV) and adeno-associated viral (ssAAV or scAAV) stocks diluted in medium were used to inoculate HepG2-NTCP cells. Sixteen hours later, viral inoculum was replaced by freshly prepared 2% DMSO-containing medium and the cells were further incubated at 37 °C for different lengths of time, as indicated in each figure legend. Viral transduction efficiency was determined by mCherry fluorescence analysis and nucleic acid quantitation as described below.

### 2.8. Visualization of Cellular RNA Synthesis by Metabolic Labeling with Ethyl Uridine

HepG2-NTCP cells were plated at a density of 4 × 10^4^ cells per well in a 96-well plate format. The day after, the medium was replaced by a 2% DMSO-containing complete medium. Twenty-four hours later, cells were treated with 2.5 μM of CPT, 50 μM of Eto, 1.25 μM of actinomycin D (as a control of RNA synthesis inhibition) or vehicle (DMSO; as control) for one hour, after which they were metabolically labeled with 0.5 mM ethyl uridine (EU). After 30 min of labeling, the medium was discarded, cells were washed and the incorporation of EU into the nascent RNA was revealed in situ after cell fixation and through a click-chemistry reaction using the Click-iT RNA HCS Assay kit (cat nº: C10327, Invitrogen), following manufacturer’s instructions. The fluorescence signal was then imaged and pictures were taken in a SparkCyto plate reader as indicated below.

### 2.9. Fluorescence Image Acquisition and Analysis

HepG2-NTCP cells plated in a 96-well plate format were subjected to compound treatment followed by vector viral transduction as indicated above. At the indicated times after transduction, the culture medium was discarded, cells were washed with PBS and fixed by incubation in 4% FA-PBS solution for ten minutes at room temperature. Cells were then extensively washed with a solution of 0.3% Triton X-100 in PBS for one hour to remove the fixative and they were incubated for 30 min with a 1xPBS solution containing a 0.5 μg/mL Hoechst 33342 dye. Viral vector-derived mCherry, γH2AX (in immunofluorescence experiments), EU labeling and nuclei fluorescence staining were imaged and pictures were taken in a SparkCyto plate reader after extensive washes with PBS. Images of 2456 × 2052 pixels at a 16-bit gray scale were acquired with a 10× objective. All images were taken with exactly the same exposure settings. The Fiji/ImageJ v1.0 software analysis package [49] was used to quantitate the number of nuclei and mCherry-positive cells, and the mean intensity signal of each image.

### 2.10. Nucleic Acid Analysis

HepG2-NTCP cells plated in a 12-well plate format were subjected to compound treatment followed by vector viral transduction as indicated above. Forty-eight hours later, the medium was discarded, cells were washed once with PBS and nucleic acids were extracted as explained below. 

Total RNA was extracted following the guanidinium isothiocyanate extraction protocol as previously described [50]. In total, 2 μg of total RNA purified from each sample was subjected to DNase treatment (dsDNase; cat. nº: 15205063, Fisher Scientific, Waltham, MA, USA) in a final volume of 10 μL, following the manufacturer’s instructions. Half of the material was used to quantify mCherry and GAPDH mRNA (for normalization) in each sample in a two-step RT-qPCR assay using the MultiScribe Reverse Transcriptase (cat. nº: 4319983, Applied Biosystems, Waltham, MA, USA) and the PowerTrack^TM^ SYBR Green qPCR Master Mix (cat. nº: A46109, ThermoFisher Scientific, Waltham, MA, USA). Next, 10-fold serial dilutions of plasmids containing each target sequence were prepared as standard curves to be used with each corresponding pair of primers: mCherry (5′-TTCATGTACGGCTCCAAGGC-3′ and 5′-TGTAGATGAACTCGCCGTCC-3′) and GAPDH (5′-TGGAAGATGGTGATGGGATTT-3′ and 5′-AGGTGAAGGTCGGAGTCAACG-3′). The results were normalized using GAPDH mRNA levels in each sample and were displayed in the figures as the number of copies per 100 ng of total RNA.

Total DNA was extracted using the NZY Tissue gDNA Isolation kit (cat. nº: MB13503, NzyTech) following the manufacturer’s instructions. The RNase treatment step was included right after sample lysis to make sure RNA-free DNA was obtained. Next, 5 ng of purified RNA-free DNA from each sample was used for direct quantitation of total mCherry and GAPDH DNA content (for normalization) by qPCR using the PowerTrack^TM^ SYBR Green qPCR Master Mix. Then, 10-fold serial dilutions of plasmids containing each target sequence were prepared as standard curves to be used with each corresponding pair of primers: mCherry (same primers as the ones used in mCherry RT-qPCR, above) and GAPDH (5′-CATGGTGCCAAGCCGGGAGA-3′ and 5′-GGGTCGGGTCAACGCTAGGC-3′).

DNA integration was analyzed using an in-house optimized two-step PCR protocol. First, 5 ng of purified RNA-free DNA was subjected to a PCR protocol using the Speedy NZYTaq 2× Green Master Mix and an mCherry specific primer (Cherry1Alu: 5′-GTGAGCAAGGGCGAGGAGGATAACATGGC-3′) together with an AluII specific primer (5′-GCCTCCCAAAGTGCTGGGATTACAG-3′). The PCR conditions were: 1 cycle at 95 °C for 1 min; 20 cycles of 2 s at 94 °C, 5 s at 60 °C and 25 s at 72 °C; and 1 cycle at 72 °C for 2 min. This first PCR allows the amplification of mCherry integrated events. Next, 2.5 μL of 1:10 diluted PCR products was used as templates in a nested-qPCR assay using the same qPCR conditions and mCherry primers described above for total mCherry DNA and mCherry mRNA analysis. 

### 2.11. Statistical Analysis

GraphPad Prism v.10.0.0 software was used to analyze the data, prepare the graphs and perform the statistical analysis. All experimental results (immuno-/fluorescence intensity analysis, MTT assays, DNA and RNA analysis) are displayed in the graphs as the mean ± standard deviation (S.D.). Each experiment was performed at least two times in triplicate wells as stated in each figure legend. Normal distribution of the data was confirmed using the Shapiro–Wilk normality test. For data distributed normally, the differences among the means between multiple groups (more than two) were analyzed by one-way ANOVA followed by Dunnett’s multiple comparison test, as indicated in each figure legend. Data not distributed normally were analyzed by a nonparametric Kruskal–Wallis test followed by Dunn’s multiple comparison test, as indicated in each figure legend. The statistical significance was set as: ns, not significant; * *p* < 0.05; ** *p* < 0.01; *** *p* < 0.001; **** *p* < 0.0001. 

## 3. Results

### 3.1. Transient Camptothecin and Etoposide Treatment Induce DDR Activation without Affecting Cell Viability

It is well known that inhibition of cellular topoisomerases induces double-strand breaks in cellular chromosomal DNA and activates DNA damage response (DDR) pathways in a wide variety of cell lines [34]. In agreement with that, treatment of HepG2-NTCP hepatic cells with CPT and Eto induced DDR activation, as determined by the immunofluorescence detection of histone H2AX phosphorylation, a marker of DNA damage. As shown in Figure S1A, H2AX phosphorylation readily increased in CPT- and Eto-treated cells compared to the vehicle conditions. In general, the phenotypic effect was more pronounced in Eto-treated cells compared to CPT, since it reached a higher proportion of cells and displayed a clear dose–response effect (Figure S1B). These treatment conditions did not have a measurable impact on cell viability in the time frame of the experiment as determined by MTT assays performed in parallel (Figure S1C). Based on the impact of CPT and Eto in H2AX phosphorylation and cell viability, we selected 2.5 μM of CPT and 50 μM of Eto as the experimental conditions for the following experiments. 

**Figure 1:**
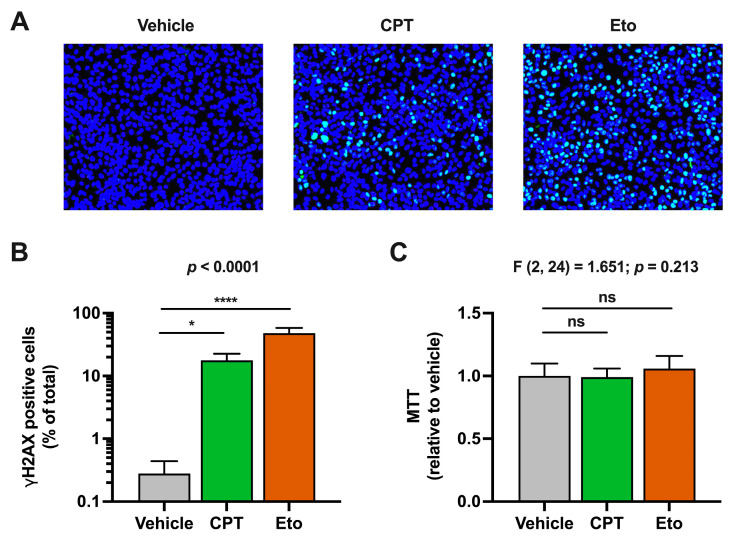
Camptothecin (CPT) and etoposide (Eto) treatment induce DNA damage response (DDR) without affecting cell viability. Cells were treated for one hour with either vehicle, 2.5 μM of CPT or 50 μM of Eto. H2AX phosphorylation (γH2AX) was detected by immunofluorescence analysis and MTT assays were performed to measure cell viability. (**A**) Representative images of the overlay of γH2AX staining (in green) and cell nuclei (in blue) are shown. (**B**) Quantitation of the percentage of γH2AX positive cells relative to the total number of cells in each experimental condition. (**C**) Cell viability (MTT assays) results relative to vehicle-treated cells that were set as 1. Data displayed in graphs are the mean values and standard deviation of three independent experiments performed in triplicates (*n* = 9). Data from (**B**) were not normally distributed and were analyzed using nonparametric Kruskal–Wallis test followed by Dunn’s multiple comparisons test. Instead, data from (**C**) were normally distributed and therefore, they were analyzed using one-way ANOVA followed by Dunnett’s post-hoc test. Statistical information is shown above each graph. Dunnett’s and Dunn’s tests were used to determine the statistical significance in pairwise comparisons (n.s.: not significant; * *p* < 0.05; **** *p* < 0.0001).

As shown in Figure 1, we barely detected H2AX phosphorylation in the vehicle-treated cells, suggesting that non-dividing hepatic cells do not suffer considerable DNA damage under basal conditions or they repair it very rapidly. Importantly, H2AX phosphorylation was detected in ~10% of CPT- and ~40% of Eto-treated cells, under these experimental conditions.

As previously mentioned, to ensure that CPT and Eto treatments did not compromise cell viability, MTT assays were performed after compound treatment. As shown in Figure 1C, no statistically significant differences were observed in compound-treated cells compared to the control conditions. All these results confirmed that CPT and Eto induce DNA damage responses in hepatic HepG2-NTCP cells without affecting cell viability.

### 3.2. Transient Camptothecin and Etoposide Treatment Increase rAAV Transduction Efficiency

Next, we evaluated the impact of topoisomerase inhibitors on epichromosomal DNA expression. To do so, we chose rAAV vectors because they are able to transduce non-dividing hepatocytes and deliver their DNA genome that remains in an epichromosomal state in the nucleus of the cells. In this study, we used two types of rAAV vectors: ssAAV that delivers an ssDNA genome that needs to be converted into dsDNA prior to its expression, and an scAAV that directly delivers a dsDNA template. Both types of AAV vectors express a nuclear-tagged mCherry reporter protein, which allows the easy detection of individual transduction events in hepatic cells.

Transduction efficiency was analyzed by quantitation of the percentage of mCherry-positive cells and the mean fluorescence intensity signal upon transduction at a wide-range multiplicity of infection (MOI) conditions (i.e., from 50 to 12,800 vg/cell). Transduction with scAAV yielded a higher percentage of mCherry-positive cells compared to ssAAV transduction in every MOI condition tested (Figure S2). These results are consistent with previously published results [13], demonstrating that scAAV-derived transduction is more efficient than that of ssAAV because dsDNA conversion is a limiting step for epichromosomal AAV expression in hepatic cells.

**Figure 2:**
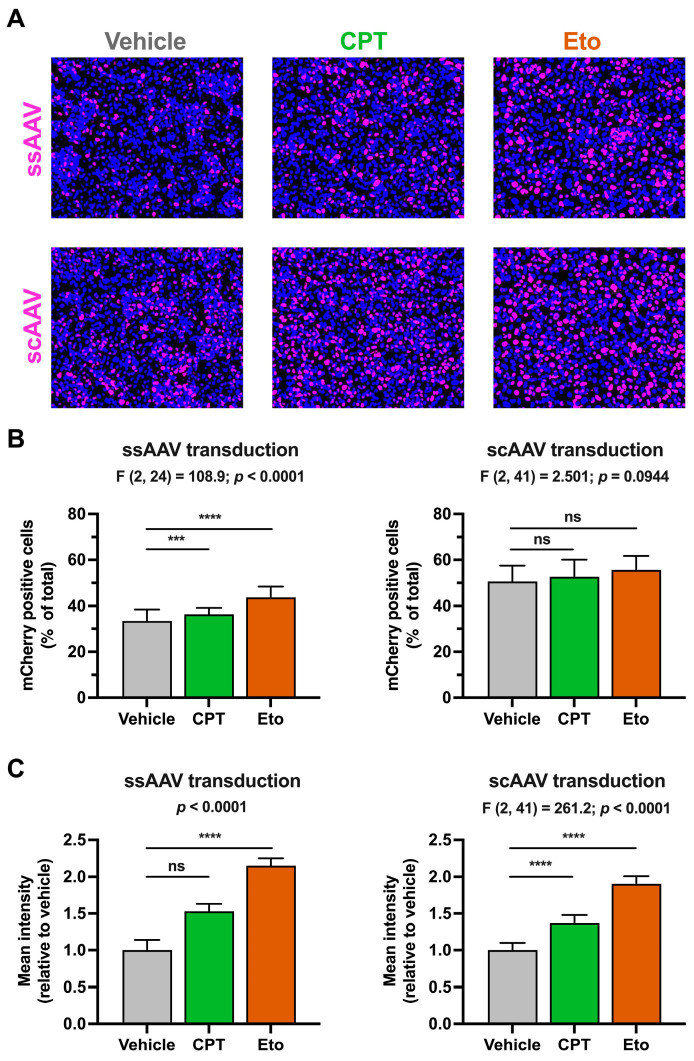
Camptothecin and etoposide increase rAAV transduction. Cells were treated for one hour with either vehicle, 2.5 μM of CPT or 50 μM of Eto, and then transduced with ssAAV (2500 vg/cell) or scAAV (100 vg/cell). mCherry accumulation was measured 4 days after. (**A**) Representative overlay images of the mCherry accumulation (magenta) and nuclei staining (blue) in ssAAV- (upper panels) and scAAV- (lower panels) transduced cells. (**B**) Quantitation of the percentage of mCherry-positive cells relative to the total number of cells in each experimental condition. Results from ssAAV-transduced cells are shown on the left graph, while results from scAAV-transduced cells are on the right graph. (**C**) Quantitation of the mCherry mean fluorescence intensity signal in each condition relative to vehicle-treated condition that was set as 1. Results from ssAAV-transduced cells are shown on the left graph, while results from scAAV-transduced cells are on the right graph. Data displayed in graphs are the mean values and standard deviation of three (in ssAAV) or five (in scAAV) independent experiments performed in triplicates (*n* = 9 or *n* = 15). Data from (**B**,**C**) (only right graph) that were normally distributed were analyzed using one-way ANOVA followed by Dunnett’s post-hoc test. Instead, data shown in the left graph in panel C were not normally distributed and therefore, they were analyzed using nonparametric Kruskal–Wallis test followed by Dunn’s multiple comparisons test. Statistical information is shown above each graph. Dunnett’s and Dunn’s tests were used to determine the statistical significance in pairwise comparisons (n.s.: not significant; *** *p* < 0.001; **** *p* < 0.0001).

In order to study the effect of topoisomerase inhibitors on rAAV-derived gene expression and considering the abovementioned efficiency differences between the two types of vectors, we adjusted the MOIs used in each type of rAAV transduction so that similar mCherry levels would be produced in vehicle-treated conditions after 4 days of transduction (i.e., ssAAV at 2500 vg/cell and scAAV at 100 vg/cell). As shown in Figure 2A and quantitated in Figure 2B, transduction with ssAAV and scAAV produced measurable mCherry signals in around 33% and 51% of cells, respectively. CPT and Eto treatments slightly increased the percentage of mCherry-positive cells upon ssAAV (CPT: 36%; Eto: 44%) and scAAV (CPT: 53%; Eto: 56%) transduction. These differences were statistically significant in ssAAV transduction conditions, but not in scAAV-transduced cells. Importantly, both CPT and Eto treatments produced a 1.5- to 2-fold increase in the mCherry mean fluorescence intensity signal in both rAAV transduction systems. 

Interestingly, Eto treatment produced a stronger increase in mCherry accumulation compared to CPT treatment in both types of rAAV. This difference was already detectable by day 2 post-transduction and increased over time as shown in Figure S3. Dose–response experiments demonstrated that a further increase in CPT and Eto concentration produces a small but statistically significant increase in the mCherry mean fluorescence intensity signal (Figure S4). Collectively, these results demonstrate that CPT and Etoposide pretreatment increase rAAV transduction regardless of the type of AAV used, suggesting that the effect is independent of the second strand DNA synthesis during the AAV genome strand repair process and probably occurs downstream of this step. These results are compatible with the hypothesis that the inhibition of topoisomerases may increase incoming epichromosomal DNA expression.

### 3.3. Transient Camptothecin and Etoposide Treatment Increase IDLV Transduction Efficiency

To further investigate the effect of CPT and Eto treatment on epichromosomal DNA expression, we took advantage of a different epichromosomal DNA viral vector known as integration deficient lentivirus (IDLV) [24]. IDLVs are recombinantly produced lentiviruses that carry point mutations in the active site of the viral integrase protein rendering these viruses unable to integrate their DNA genome into the cellular chromosomal DNA by a canonical integrase-mediated process. Instead, their genomes are repaired into 1-LTR or 2-LTR containing circular DNA molecules that are kept in epichromosomal form in the nuclei of cells and are responsible for viral gene expression. Therefore, we transduced vehicle-, CPT- and Eto-treated cells with IDLVs expressing the same nuclear-tagged mCherry reporter gene used in the rAAV systems. In parallel, we also performed transduction experiments with ICLV, in order to determine whether these compounds specifically affect epichromosomal DNA expression or instead have a broader effect on incoming DNAs irrespective of their intrinsic ability to integrate their genomes into the host chromosomal DNA. 

We first compared IDLV and ICLV transduction efficiencies in the absence of treatment by performing titration assays and quantitating the mCherry accumulation four days later. ICLV transduction was more efficient compared to its IDLV counterpart as determined by the percentage of mCherry-positive cells and the mean fluorescence intensity signal (Figure S5). These results agree with previous reports and with the fact that lentiviral expression strongly depends on viral genome integration into the host chromosomal DNA [27]. 

**Figure 3:**
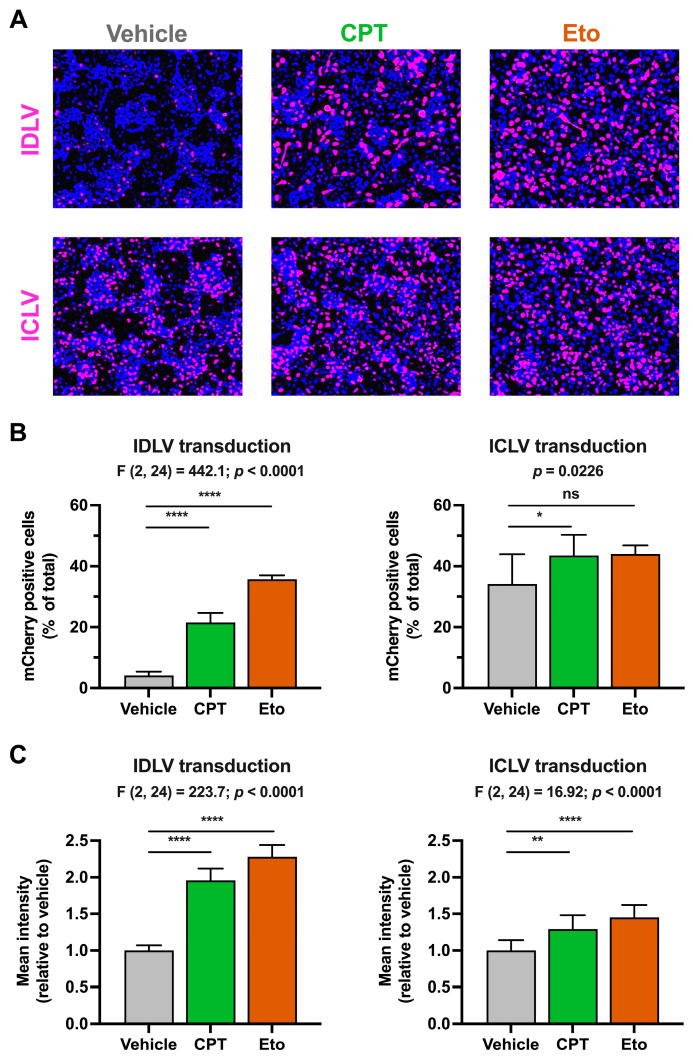
Camptothecin and etoposide increase IDLV transduction. Cells were treated for one hour with either vehicle, 2.5 μM of CPT or 50 μM of Eto, and then transduced with IDLV (25 μL/well) or ICLV (3 μL/well). mCherry accumulation was measured 4 days after. (**A**) Representative overlay images of mCherry accumulation (magenta) and nuclei staining (blue) in IDLV- (upper panels) and in ICLV- (lower panels) transduced cells. (**B**) Quantitation of the percentage of mCherry-positive cells relative to the total number of cells in each experimental condition. (**C**) Quantitation of the mCherry mean fluorescence intensity signal in each condition relative to the vehicle-treated condition that was set as 1. Results from IDLV-transduced cells are shown on the left graphs, while the results from ICLV-transduced cells are on the right graphs. Data displayed in graphs are the mean values and standard deviation of three independent experiments performed in triplicates (*n* = 9). Data from (**B**) (only right graph) and (**C**) that were normally distributed were analyzed using one-way ANOVA followed by Dunnett’s post-hoc test. Instead, data shown in the right graph in panel B were not normally distributed and therefore, they were analyzed using nonparametric Kruskal–Wallis test followed by Dunn’s multiple comparisons test. Statistical information is shown above each graph. Dunnett’s and Dunn’s tests were used to determine the statistical significance in pairwise comparisons (n.s.: not significant; * *p* < 0.05; ** *p* < 0.01; **** *p* < 0.0001).

As previously performed in the rAAV system, we first treated the cells with CPT and Eto and then transduced them with doses of lentiviruses that allow the detection of mCherry four days later in both IDLV and ICLV systems. As shown in Figure 3, CPT and Eto pre-treatments produced an increase in the number and the mean fluorescence intensity signal of mCherry-positive cells in IDLV-transduced cells. The magnitude of these effects was more pronounced in Eto- than in CPT-treated conditions, reaching up to 35% of positive cells in Eto-treated cells compared to 21% in CPT and 4% in vehicle cells. In contrast to the strong effect observed in IDLV-mCherry expression, CPT and Eto treatments produced a very modest increase (from 35% in vehicle to 43% and 45% in CPT- and Eto-treated conditions, respectively) in ICLV-mCherry expression.

The difference in the magnitude of the effect observed upon IDLV and ICLV transduction suggests that the effect of topoisomerase inhibitors is specific, or at least more effective, in non-integrated epichromosomal DNAs. A small but statistically significant increase in the mCherry mean fluorescence intensity was observed upon CPT and Eto treatment in ICLV-transduced cells. This increase is compatible with the fact that 1-LTR and 2-LTR containing episomal DNAs can also be produced upon ICLV transduction [25], albeit in much lower amounts compared to integrated ICLV DNA forms [27].

Dose–response experiments performed with wide-range concentrations of CPT and Eto demonstrated statistically significant increases in the number and mean fluorescence intensity signal of mCherry-positive cells in most of the experimental conditions (Figure S6). Kinetic analysis of the effect of compound treatment on mCherry reporter accumulation showed that the increase in expression occurs from very early time points after both IDLV and ICLV transduction (Figure S7). However, while in the IDLV system the differences were bigger in magnitude and were maintained over time, in the ICLV system, the maximal difference peaked at day 2 and then it declined over time. These results suggest that the kinetics of IDLV and ICLV transduction upon CPT and etoposide treatments are different. Taken together, all these results demonstrate that CPT and Eto treatments increase the expression of epichromosomal DNAs, such as rAAV or IDLV genomes.

### 3.4. Transient Camptothecin and Etoposide Treatment Increase IDLV-Derived mRNA Accumulation

To further characterize the mechanism by which CPT and Eto treatments increase IDLV transduction, we quantitated viral DNA and mRNA by qPCR and RT-qPCR, respectively, in both LV strains. In contrast to previous experimental set ups where lentiviral doses had to be adjusted to allow the detection of mCherry in basal conditions from both strains, for this new set of experiments, we transduced cells with equal amounts of both strains to ensure that the cells received the same amount of input viral genomes. Since viral DNA and mRNA production precede mCherry protein accumulation and given that phenotypic effects were observed at early time points (see Figure S7), samples for nucleic acid analysis were harvested 48 h after transduction.

**Figure 4:**
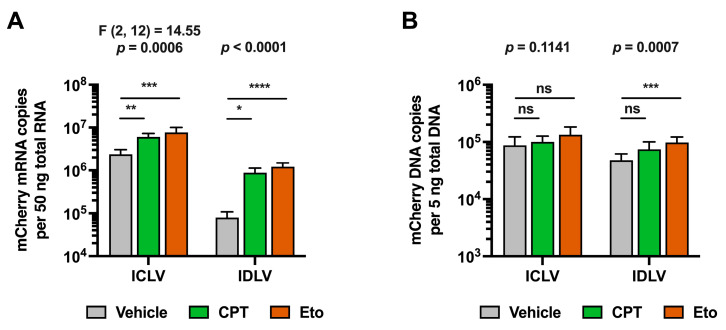
Camptothecin and etoposide increase IDLV-derived mCherry mRNA accumulation. Cells were treated for one hour with either vehicle, 2.5 μM of CPT or 50 μM of Eto, and then transduced with equal amounts of IDLV or ICLV (250 μL/well in 12-well plate format). Nucleic acids were extracted from cell lysates 48 h later. (**A**) Quantitation of mCherry reporter mRNA copy number in ICLV- or IDLV- transduced cells by RT-qPCR. (**B**) Quantitation of mCherry viral genome copy number in ICLV- or IDLV-transduced cells by qPCR. Data displayed in graphs are the mean values and standard deviation of five to nine replica distributed in 2 to 3 independent experiments (*n* = 5–9). Data from ICLV transduction in panel A that were normally distributed were analyzed using one-way ANOVA followed by Dunnett’s post-hoc test. Instead, data from IDLV transduction in panel A and data in panel B were not normally distributed, and therefore, they were analyzed using nonparametric Kruskal–Wallis test followed by Dunn’s multiple comparisons test. Statistical information is shown above each graph. Dunnett’s and Dunn’s test were used to determine the statistical significance in pairwise comparisons (n.s.: not significant; * *p* < 0.05; ** *p* < 0.01; *** *p* < 0.001; **** *p* < 0.0001).

Despite differences in the experimental set up, increased mCherry accumulation was also observed in these experiments in IDLV-transduced cells compared to ICLV transduction upon CPT and Eto treatments 48 h after LV inoculation (Figure S8). This phenotypic effect was confirmed at the mRNA level. As expected, and shown in Figure 4A, ICLV transduction produced around 30-fold more mCherry mRNA than IDLV transduction. CPT and Eto treatments produced an increase in mCherry mRNA levels in both IDLV and ICLV-transduced cells. However, differences in the magnitude of increase existed between different LVs. While CPT increased mCherry mRNA accumulation by 12-fold in IDLV-transduced cells, it barely induced a 2-fold increase in ICLV-transduced cells. Similarly, Eto treatment increased mCherry mRNA levels by 18-fold in IDLV-transduced cells but only produced a 3-fold increase in the ICLV system. Importantly, these differences in magnitude could not be explained by differences in the LV DNA copy number. As shown in Figure 4B, the viral genome copy numbers in the different conditions remained within a 2-fold range, ruling out the possibility that steps prior to lentiviral DNA production (e.g., viral entry or reverse transcription) are affected by CPT and Eto treatments.

These results demonstrate that CPT and Eto increase IDLV expression by regulating a step downstream of viral vector DNA synthesis, suggesting that they might differentially affect epichromosomal vs. integrated DNA gene expression.

### 3.5. Transient Camptothecin and Etoposide Treatment Does Not Increase General Cellular RNA Synthesis

**Figure 5:**
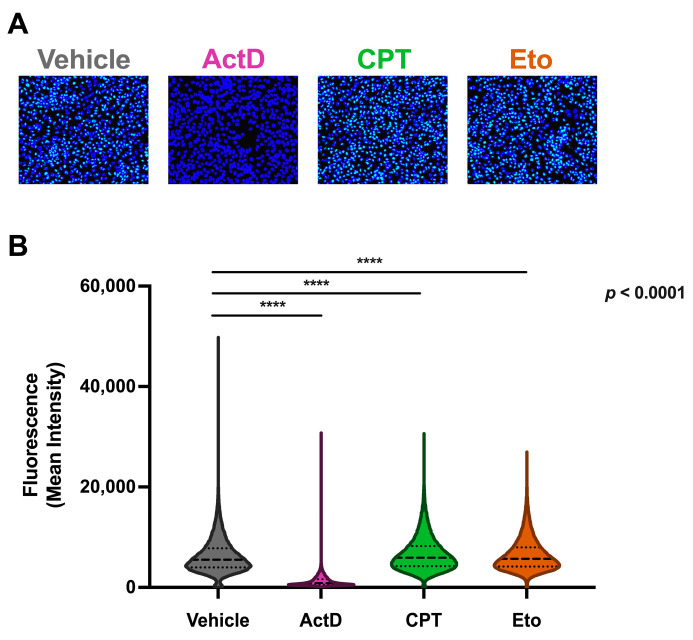
Campothecin and etoposide treatments do not increase global cellular RNA synthesis. Cells were treated for one hour with either vehicle, 2.5 μM of CPT, 50 μM of Eto or 1.25 μM of actinomycin D (ActD), and then further incubated for 30 min with a 0.5 mM ethyl uridine (EU)-containing media. Cells were then fixed and subjected to a click chemistry labeling reaction protocol for analysis. (**A**) Representative overlay images of EU labeling (green) and nuclei staining (blue) in vehicle-, ActD-, CPT- and Eto-treated cells. (**B**) Quantitative analysis of the mean fluorescence intensity of individual cells in each condition. Data are displayed as violin plots. Each violin represents the frequency (width) of the mean fluorescence intensity per cell. Data displayed contain values from three independent experiments performed in triplicates and were analyzed using nonparametric Kruskal–Wallis test followed by Dunn’s multiple comparisons test (**** *p* < 0.0001).

Since IDLV gene expression is dependent on cellular RNA polymerase II (RNAPII) activity and in order to determine whether the increased episomal DNA expression upon CPT and Eto treatments was not due to an unspecific effect on cellular RNA synthesis, we analyzed the impact of topoisomerase inhibitors on cellular RNA production. To do so, we took advantage of ethyl-uridine (EU) click chemistry technology. This technology allows the visualization and quantitation of nascent RNA through the conjugation of a fluorescent dye in fixed cells, after brief metabolic pulse labeling with a modified nucleotide analog. EU-labeling was performed in vehicle-, CPT- and Eto-treated cells. As a control, we treated cells with a well-known cellular RNA polymerase inhibitor: actinomicin D (ActD). As shown in Figure 5, treatment with ActD completely prevented EU incorporation, demonstrating the specificity of this technique to detect and quantify cellular transcription in HepG2-NTCP cells. Importantly, we did not observe any increase in cellular RNA synthesis upon treatment with CPT or Eto compared to vehicle-treated cells, suggesting that the effect of CPT and Eto treatment on the epichromosomal gene expression (e.g., AAV and IDLV) is specific and cannot be explained by a general increase in RNAPII activity.

### 3.6. Transient Camptothecin and Etoposide Treatment Increase IDLV Integration

After ruling out the possibility that topoisomerase inhibitors increased cellular RNA synthesis, we compared the effect of CPT and Eto treatments on the integration rate of IDLV and ICLV genomes. To do so, we measured the number of integrated viral genomes in IDLV- and ICLV-transduced cells by a two-step amplification method based on an Alu-PCR followed by a nested-qPCR. Alu-PCR is a well-established methodology to measure lentiviral DNA integration [51,52,53]. This technique is based on the amplification by PCR of chimeric DNA sequences composed by both host and viral sequences using a specific pair of primers, one of which targets the Alu repeated sequences present in the host genome, and the second one that targets the viral genome. This first PCR yields a DNA product largely heterogeneous in size that is subsequently used as a template in a nested qPCR where primers specific for the mCherry sequence are used. 

**Figure 6:**
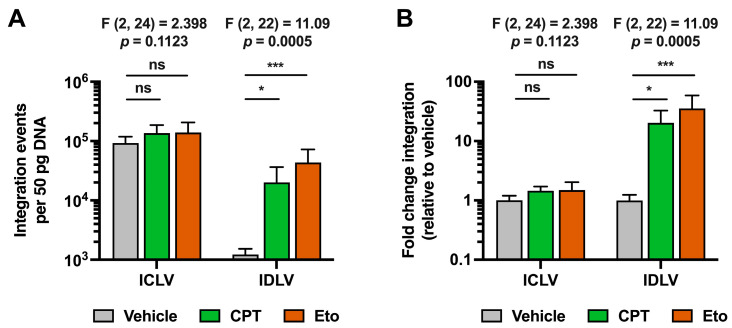
Camptothecin and etoposide treatments increase IDLV viral genome integration into cellular DNA. Cells were treated for one hour with either vehicle, 2.5 μM of CPT or 50 μM of Eto, and then transduced with equal amounts of IDLV or ICLV (250 μL/well in 12-well plate format). Nucleic acids were extracted from cell lysates 48 h later. (**A**) Quantitation of total integration events in 50 pg of total cellular DNA. (**B**) Representation of number of integration events relative to vehicle control condition for each of the lentiviruses. Data displayed in graphs are the mean values and standard deviation of three independent experiments performed in triplicates (*n* = 9). Since all data were normally distributed, they were analyzed using one-way ANOVA followed by Dunnett’s post-hoc test. Statistical information is shown above each graph. Dunnett´s test was used to determine the statistical significance in pairwise comparisons (n.s.: not significant; * *p* < 0.05; *** *p* < 0.001).

As shown in Figure 6, integration events were readily detectable in ICLV-transduced cells in basal conditions, while they were almost undetectable upon IDLV transduction. Since we have previously shown that IDLV and ICLV transductions produced similar levels of total viral DNA genomes, these results demonstrate that IDLV fails to integrate its viral genome into the hepatic host chromosomal DNA, as others have previously reported in other cellular systems [26]. Importantly, CPT and Eto treatment greatly increased integration events in IDLV-transduced cells compared to vehicle-treated conditions. In contrast, CPT and Eto treatments produced only a marginal increase, albeit not statistically significant, in the integration rate of ICLV genomes, demonstrating a differential effect of topoisomerase inhibitors in both types of lentiviruses. 

## 4. Discussion

Gene therapy is a promising strategy to treat and cure inherited metabolic liver disorders [11]. Expression of the wild-type form of a mutated allele in the affected tissues can correct a disorder and, depending on the disease, achieve life-long benefits. To do so, systemic administration of high vector doses is sometimes required to achieve therapeutic levels in solid organs, such as the liver. This may induce the appearance of undesired adverse events that need to be overcome. This has motivated the search for more effective viral vectors and also the use of small molecules that could act as adjuvants [32,33]. However, the use of small molecules may bring with it the appearance of new risks, such as those derived from an increased integration of viral genome used for therapy. This justifies the need to evaluate the risks derived from the use of compounds during viral vector transduction.

In this study, we demonstrated that a very short, one hour treatment with topoisomerase inhibitors prior to viral vector transduction is enough to increase reporter expression of rAAVs as well as rLVs in hepatic cells. These viral vectors enter the cells through different cell entry receptors and express the transgene under different promoters. Since the increased reporter gene expression occurs in both viral vector transductions, we conclude that the effect of topoisomerase inhibitor treatment is independent of the entry pathway, the promoter and the viral vector used. These results suggest that topoisomerase inhibitors used in this study target a common step or feature that is shared in both viral vector transductions. Given that the compounds were added prior to viral vector transduction, it is unlikely that the observed effect is a direct consequence of the compounds on viral particles themselves or on the delivery of their genomes. Instead, we favor the hypothesis that the compounds act directly on the host cell by predisposing it in a way that vector genome expression is enhanced. In this regard, we demonstrated that cell viability and host RNA biogenesis are unaffected by the treatments, ruling out the possibility that the increased viral vector expression is a consequence of toxic effects or increased general cellular gene expression. Instead, both compounds increased the phosphorylation of histone H2AX, a hallmark of DNA damage, indicating that DDR was induced upon these treatments. 

Expression from rAAV vectors depends on the formation of genome concatemers and their circularization, which produces stable transcriptionally active structures. Since DDR activation favors these processes [54], induction of DDR by treatment with topoisomerase inhibitors could explain the increased mCherry intensity signal observed upon rAAV transduction. The magnitude of the effect with Eto treatment was always higher than that of CPT. These differences might be explained by their intrinsic capacity to induce double-strand breaks or they might point to the existence of mechanistic differences between TOP1 and TOP2 in the regulation of viral vector expression. Indeed, TOP1 inhibition by administration of CPT has been proposed to increase ssAAV DNA repair [55], suggesting that TOP1 negatively regulates the establishment of functional AAV episomal DNA. However, in a different study, treatment with TOP2 inhibitors increased both ssAAV and scAAV transgene expression [33], suggesting that modulation of AAV expression by TOP2 occurs downstream of ssAAV DNA repair. These results pointed out that inhibition of topoisomerases may have differential effects on specific steps of AAV transduction depending on the inhibitor and/or the experimental conditions used. However, our results, obtained in non-dividing hepatic cell culture conditions, showed that ssAAV and scAAV expression increased in a similar way upon topoisomerase inhibitor treatment, ruling out the possibility that topoisomerase inhibitors regulated rAAV second strand DNA synthesis, a step that is already completed in the scAAV vector. Thus, topoisomerases do not seem to play a role in ssAAV genome repair in hepatic cells, in contrast to what has been previously published in heart-derived cells [55]. This discrepancy strongly suggests the existence of cell-type and/or tissue-specific differences that affect the efficiency of rAAV transduction, and that strategies aiming to increase rAAV transduction efficiency need to consider these singularities. 

In the case of rLV transduction, topoisomerase inhibitors increased both ICLV- and IDLV-derived expression, but with a much stronger increase in the IDLV transduction system. As expected, when we compared both types of lentiviruses, ICLV produced a higher reporter accumulation compared to IDLV transduction. Both LVs produced equivalent amounts of genomic DNA copy numbers; however, mRNA production was enhanced in ICLV compared to IDLV transduction, further supporting the idea that integration is necessary for optimal rLV expression [20,21,22]. For all these reasons, we hypothesized that the IDLV expression increase observed upon topoisomerase inhibitor treatment could be explained by an increase in non-specific and integrase activity independent integration of the IDLV genome, produced as a consequence of the DNA damage induced by the compound treatment. In agreement with that, treatment with CPT and Eto increased viral genome integration in both rLVs with a particularly higher impact on IDLV, supporting the idea that topoisomerase inhibitors increased episomal DNA integration, especially when viral integrase activity is abolished [35,38,39]. The increased viral genome integration correlated with a higher viral mRNA production upon treatment. Interestingly, treatment with topoisomerase inhibitors rescued IDLV-derived reporter mRNA expression to levels comparable to those observed in ICLV-transduced cells. Topoisomerase inhibition did not change the intracellular viral genome DNA copy number, discarding an effect on rLV entry, genome retro-transcription, or DNA homeostasis. This is in contrast to previously reported results derived from HIV-infected 293T cells, where CPT and Eto treatments produced an increase in the accumulation of total reverse transcription products, which are the precursors of 2-LTR episomes and the dsDNA integration template [38]. This discrepancy suggests that topoisomerases, and therefore, the inhibition of their activity, produce differential cell type-dependent effects that in turn might regulate differential rLV transduction. The smaller increase in viral genome integration observed in ICLV transduction could be explained by the fact that ICLV can also produce non-integrated episomal DNAs as those produced upon IDLV transduction. Thus, the increased integration observed in ICLV transduction upon CPT and etoposide treatment could probably be related to the effect of the compounds on non-integrated viral genomes produced during ICLV transduction. 

When comparing both compounds, Eto treatment produced a much higher effect on viral genome integration and viral mRNA production than CPT treatment. These results agree with the fact that Eto induced more DNA damage than CPT in hepatic cells. Thus, our data confirm the notion that the extent of DNA damage induced by these compounds determines the rate of episomal DNA integration and therefore its expression [39]. This is especially important for IDLV integration, since IDLV does not contain integrase activity. In contrast, ICLV integration, given the functional role of its viral integrase, does not depend on damaged DNA for its integration and expression. This agrees with the fact that ICLV integration barely increased compared to IDLV integration upon compound treatment. Taken together, these results strongly suggest that the increased IDLV viral DNA integration in the host chromosomal genome is the main mechanism by which DNA damage-inducing agents, such as CPT and Eto, produce an increase in IDLV reporter gene expression in hepatic cells. 

In summary, our study highlights the need to monitor DNA damage and undesired integration of viral episomal DNAs into the host genome when studying chemical compounds that increase viral transduction. This study has set up the necessary tools for the discovery of new compounds with the potential to increase viral vector transduction efficiency without triggering their integration into the host genome.

## Data Availability

Supporting data are available as Supplementary Material linked to this article.

## Supplementary Materials

The following supporting information can be downloaded at: https://www.mdpi.com/article/10.3390/pharmaceutics15102459/s1, Figure S1: Dose–response analysis of camptothecin (CPT) and etoposide (Eto) treatments in DDR induction; Figure S2: Transduction efficiency of ssAAV and scAAV vectors in hepatic cells; Figure S3: Analysis of camptothecin (CPT) and etoposide (Eto) pre-treatments regarding AAV expression over time; Figure S4: Dose–response analysis of camptothecin (CPT) and etoposide (Eto) treatments regarding AAV expression; Figure S5: Transduction efficiency of ICLV and IDLV vectors in hepatic cells; Figure S6: Dose–response analysis of camptothecin (CPT) and etoposide (Eto) treatments regarding IDLV and ICLV expression; Figure S7: Analysis of camptothecin (CPT) and etoposide (Eto) pre-treatments regarding IDLV and ICLV expression over time; Figure S8: Analysis of camptothecin (CPT) and etoposide (Eto) pre-treatments on IDLV and ICLV expression.
