# Supplementary material for: Topoisomerase Inhibitors Increase Episomal DNA Expression by Inducing the Integration of Episomal DNA in Hepatic Cells
Source: Pharmaceutics. 2023 Oct 13;15(10):2459. doi: 10.3390/pharmaceutics15102459 (PMC10610421; doi:10.3390/pharmaceutics15102459)
Supplement: Supplementary file 1 [file pharmaceutics-15-02459-s001.zip › pharmaceutics-2638968-supplementary.pdf]

Supplementary Figures

Supplementary Figure S1.

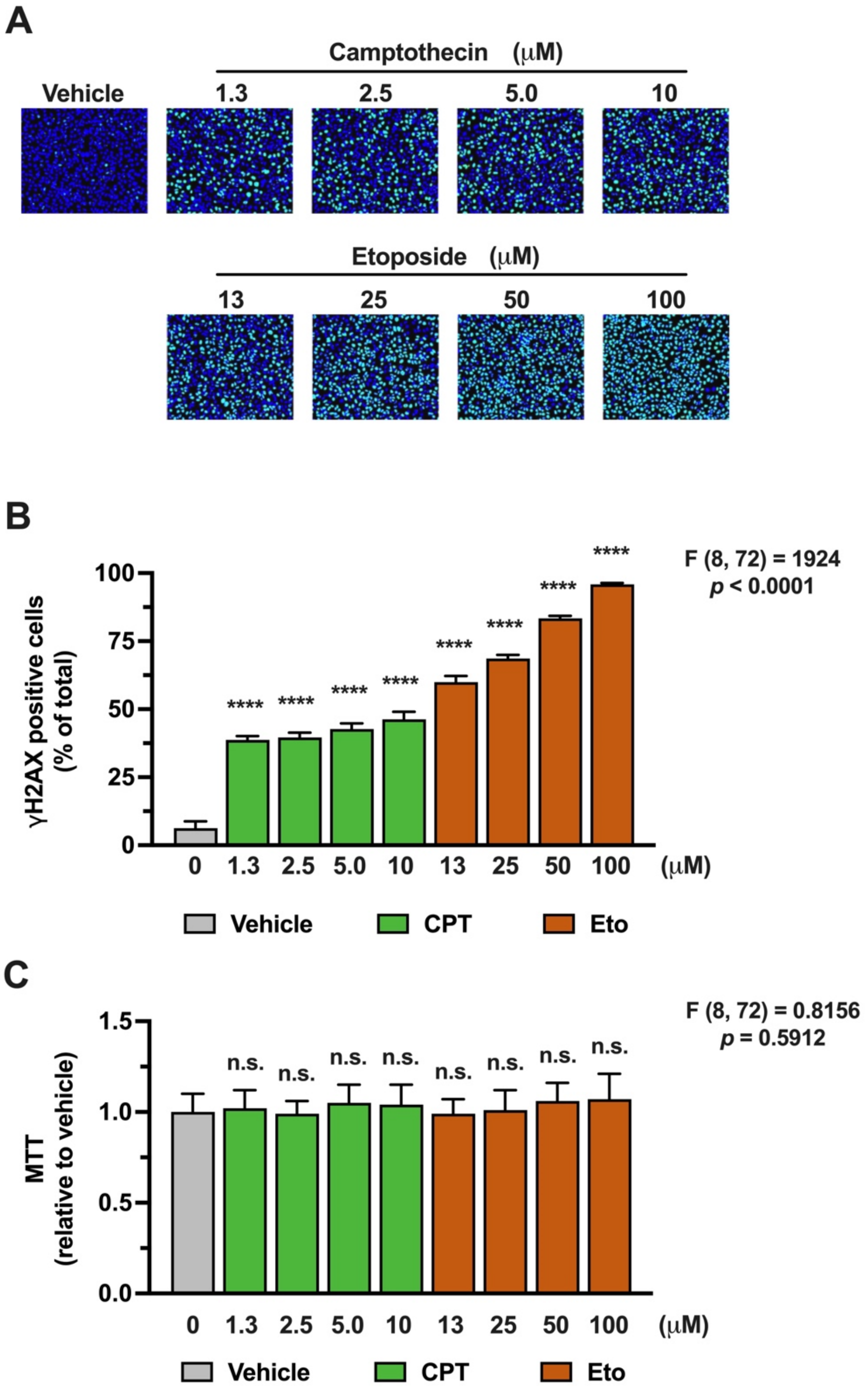

Figure S1. Dose response analysis of camptothecin (CPT) and etoposide (Eto) treatments in DNA damage response (DDR) induction. Cells were treated for one hour with a wide range of compound concentrations: vehicle, 1.25 to 10 μM of CPT, or 12.5 to 100 μM of Eto. **(A)** Representative images of the overlay of γH2AX staining (in green) and cell nuclei (in blue) is shown. **(B)** Quantitation of the percentage of γH2AX positive cells relative to the total number of cells in each experimental condition. **(C)** Cell viability (MTT assays) results relative to vehicle-treated cells that was set as 1. Data displayed in graphs are the mean values and standard deviation of three independent experiments performed in triplicates (n=9). Statistical analysis was performed using one-way ANOVA followed

by Dunnett's post-hoc test. Information about F-statistics is shown next to each graph. Dunnett´s test was used to determine the statistical significance in pairwise comparisons (n.s.: not significant; **** $P < 0.0001$). These data were used for the selection of compound concentration to be used in the following experiments.

Supplementary Figure S2.

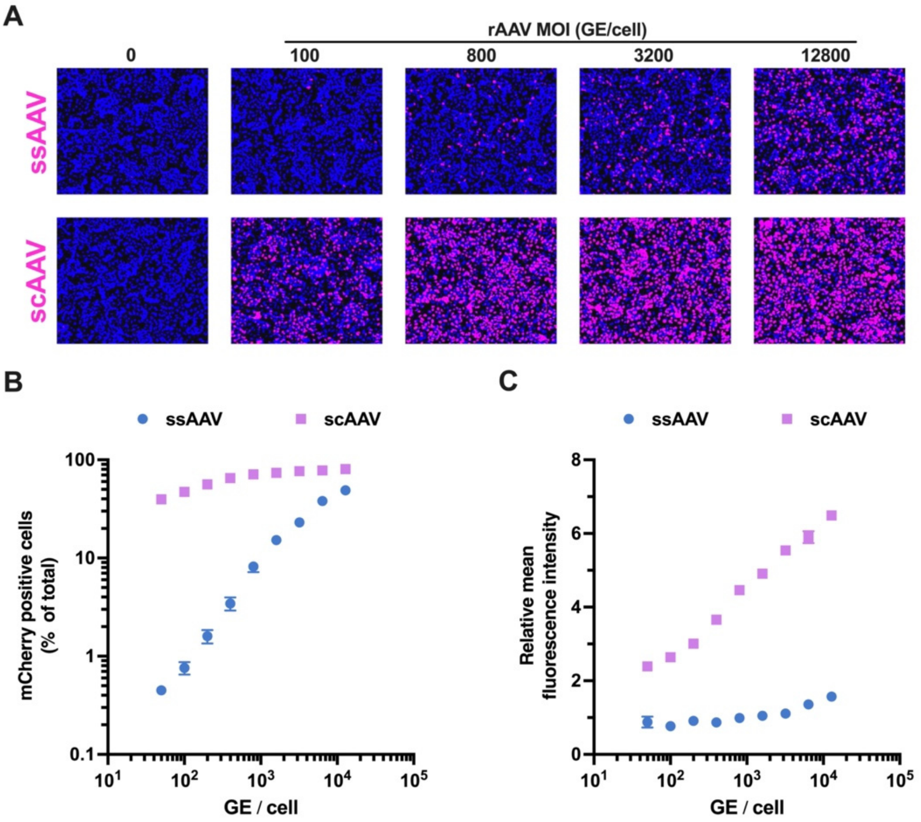

Figure S2. Transduction efficiency of single-stranded adeno-associated virus (ssAAV) and self-complementary AAV (scAAV) vectors in hepatic cells. Cells were transduced with increasing doses (multiplicity of infection (MOI) from 50 to 12.800 vg/cell) of either ssAAV-mCherry or scAAV-mCherry viral vectors. Reporter mCherry expression was analyzed 3 days later. **(A)** Representative overlay images of the mCherry fluorescence signal (in magenta) and cell nuclei staining (in blue) in selected MOI conditions are shown. **(B)** Quantitation of the percentage of mCherry positive cells relative to the total number of cells in each experimental condition. **(C)** Relative quantitation of the mCherry mean fluorescence intensity signal in each condition. Results from ssAAV-transduced cells are shown in blue circles, while results from scAAV-transduced cells are shown in pink squares. Data displayed in graphs are the mean values and standard deviation of a single experiment performed in triplicates (n=3). These data were used for the selection of ssAAV and scAAV doses to be used in the following experiments.

Supplementary Figure S3.

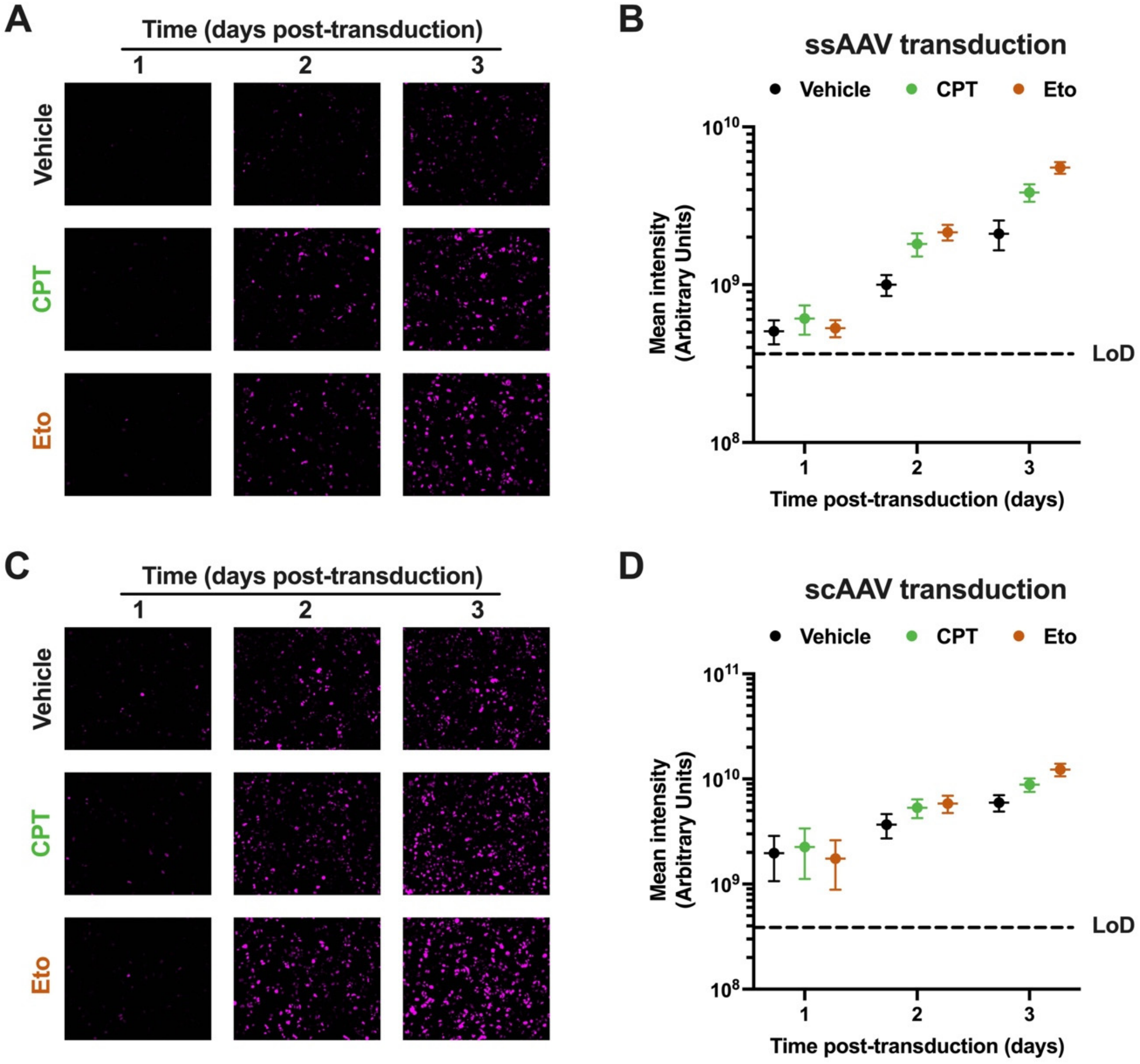

Figure S3. Analysis of CPT and Eto pre-treatments on AAV expression over time. Cells were treated for one hour with either vehicle, 2.5 µM of CPT or 50 µM of Eto and then transduced with ssAAV (2500 vg/cell) or scAAV (100 vg/cell). mCherry accumulation was measured each day during the following three days. **(A and C)** Representative images of the mCherry accumulation (magenta) in **(A)** ssAAV- and **(C)** scAAV-transduced cells are shown for each experimental condition at each time point. **(B and D)** Quantitation of the mCherry mean fluorescence intensity signal in each condition over the time frame of the experiment in shown. Data displayed in graphs are the mean values and standard deviation of three (A and B) or five (C and D) independent experiments, each one performed in triplicates (n=9 or n=15). Note that the effect of topoisomerase inhibitors on ssAAV and scAAV is already detectable by day 2 post-transduction and increases over time. These kinetic experiments support the results shown in main Figure 2.

Supplementary Figure S4.

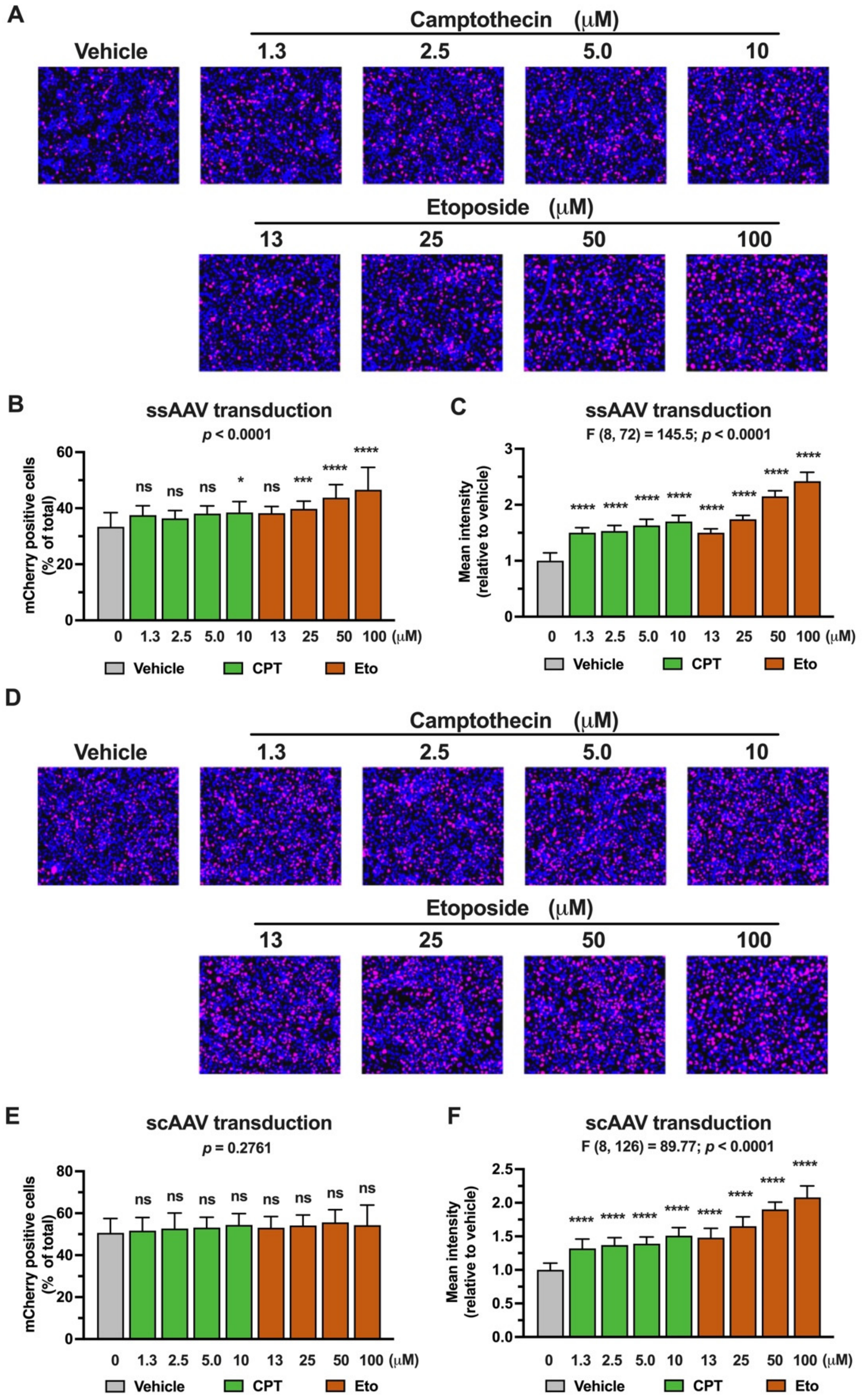

Figure S4. Dose response analysis of CPT and Eto treatments on AAV expression. Cells were treated for one hour with a wide range of compound concentrations: vehicle, 1.25 to 10 µM of CPT, or 12.5 to 100 µM of Eto and then transduced with ssAAV (2500 vg/cell) **(A-C)** or scAAV (100 vg/cell) **(D-F)**. mCherry accumulation was measured 4 days after. **(A and D)** Representative overlay images of the mCherry accumulation (magenta) and nuclei staining (blue) in ssAAV- (A) and scAAV- (D) transduced cells. **(B and E)** Quantitation of the percentage of mCherry

positive cells relative to the total number of cells in each experimental condition. **(C and F)** Quantitation of the mCherry mean fluorescence intensity signal in each condition relative to vehicle-treated condition that was set as 1. Data displayed in graphs are the mean values and standard deviation of three (A-C) or five (D-F) independent experiments, each one performed in triplicates (n=9 or n=15). Data from B and E were not normally distributed and were analyzed using nonparametric Kruskal-Wallis test followed by Dunn´s multiple comparisons test. Instead, data from C and F were normally distributed and therefore, they were analyzed using one-way ANOVA followed by Dunnett's post-hoc test. Statistical information is shown above each graph. Dunnett´s and Dunn´s tests were used to determine the statistical significance in pairwise comparisons (n.s.: not significant; * $P < 0.05$; *** $P < 0.001$; **** $P < 0.0001$). These dose response experiments support the results shown in main Figure 2.

Supplementary Figure S5.

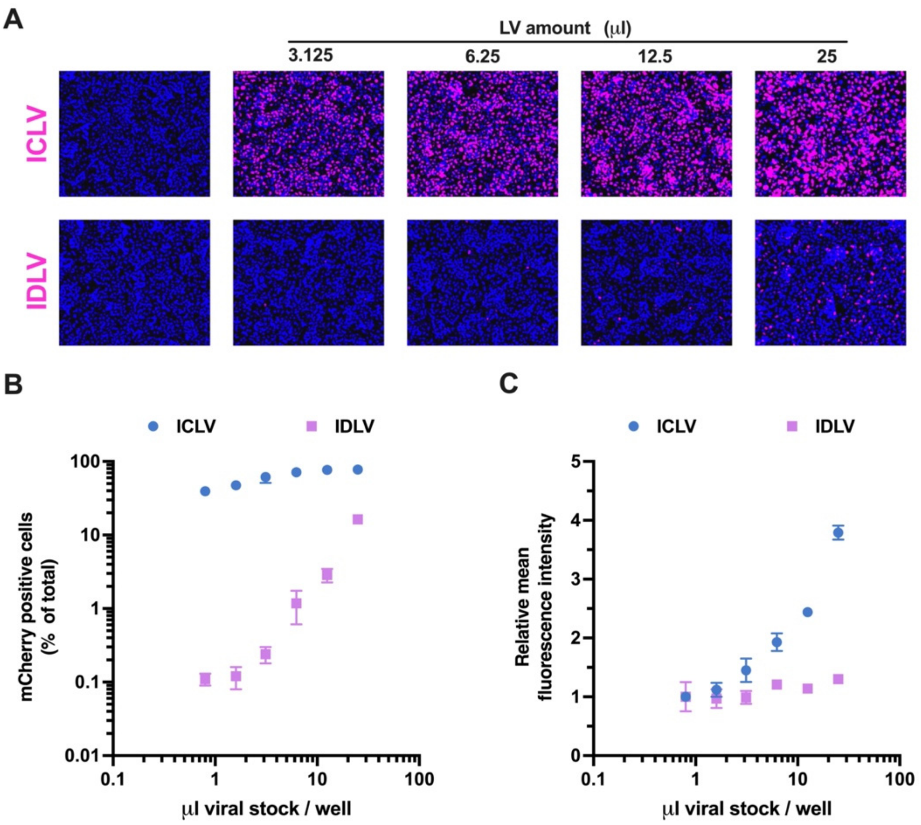

Figure S5. Transduction efficiency of integration competent lentivirus (ICLV) and integration deficient lentivirus (IDLV) vectors in hepatic cells. Cells were transduced with increasing doses (MOI from 0.78 to 25 µl virus/cell) of either ICLV-mCherry or IDLV-mCherry viral vectors. Reporter mCherry expression was analyzed 4 days later. **(A)** Representative overlay images of the mCherry fluorescence signal (in magenta) and cell nuclei staining (in blue) in selected MOI conditions are shown. **(B)** Quantitation of the percentage of mCherry positive cells relative to the total number of cells in each experimental condition. **(C)** Relative quantitation of the mCherry mean fluorescence intensity signal in each condition. Results from ICLV-transduced cells are shown in blue circles, while results from IDLV-transduced cells are shown in pink squares. Data displayed in graphs are the mean values and standard deviation of a single experiment performed in triplicates (n=3). These data were used for the selection of ICLV and IDLV doses to be used in the following experiments.

Supplementary Figure S6.

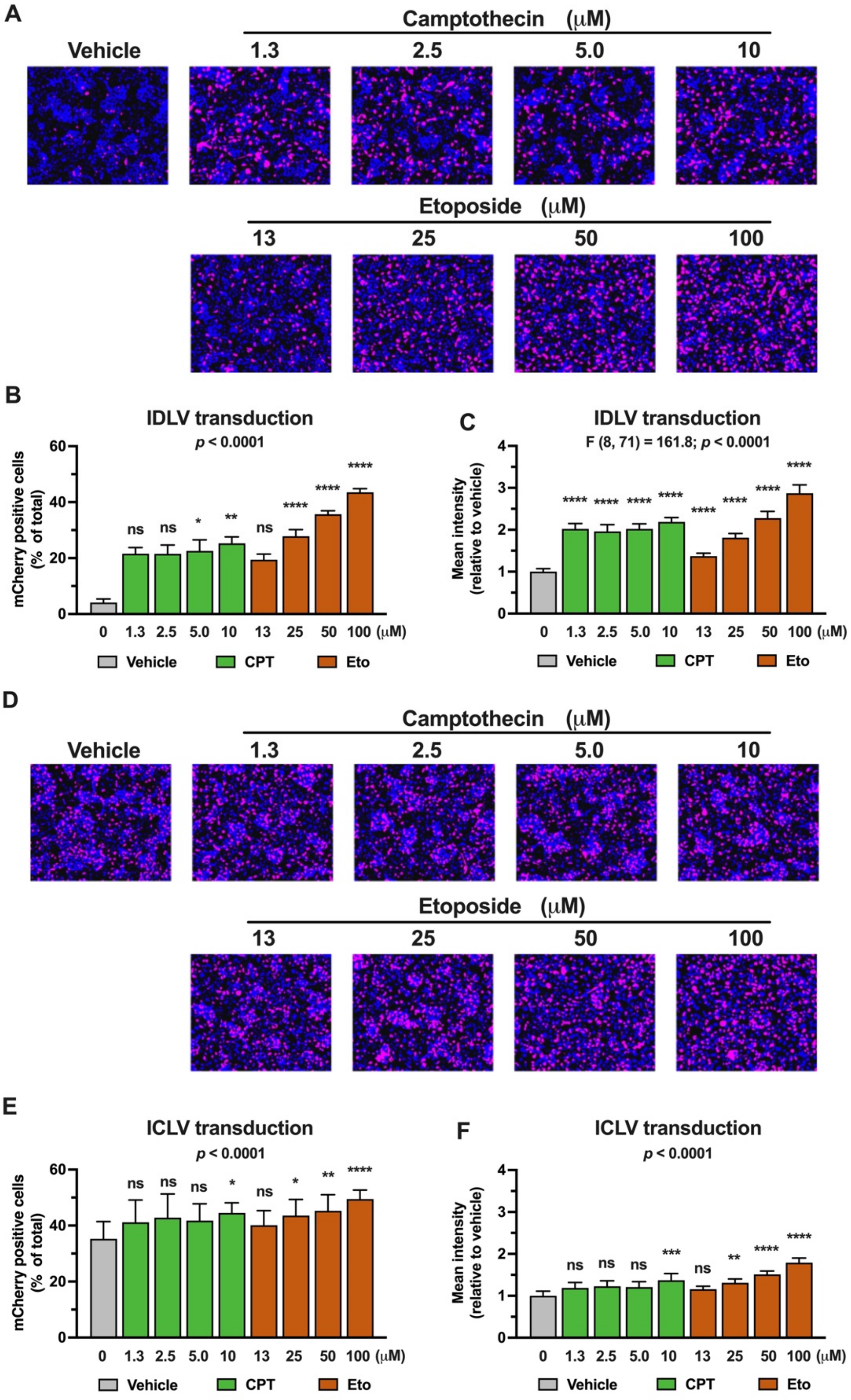

Figure S6. Dose response analysis of CPT and Eto treatments on IDLV and ICLV expression. Cells were treated for one hour with a wide range of compound concentrations: vehicle, 1.25 to 10 μM of CPT, or 12.5 to 100 μM of Eto and then transduced with IDLV-mCherry (25 μl virus/cell) **(A-C)** or ICLV-mCherry (3 μl virus/cell) **(D-F)**. mCherry accumulation was measured 4 days after. **(A and D)** Representative overlay images of the mCherry accumulation (magenta) and nuclei staining (blue) in IDLV- (A) and ICLV- (D) transduced cells. **(B and E)**

Quantitation of the percentage of mCherry positive cells relative to the total number of cells in each experimental condition. **(C and F)** Quantitation of the mCherry mean fluorescence intensity signal in each condition relative to vehicle-treated condition that was set as 1. Data displayed in graphs are the mean values and standard deviation of three independent experiments performed in triplicates (n=9). Data from B, E and F were not normally distributed and were analyzed using nonparametric Kruskal-Wallis test followed by Dunn´s multiple comparisons test. Instead, data from C were normally distributed and therefore, they were analyzed using one-way ANOVA followed by Dunnett's post-hoc test. Statistical information is shown above each graph. Dunnett´s and Dunn´s tests were used to determine the statistical significance in pairwise comparisons (n.s.: not significant; * $P < 0.05$; ** $P < 0.01$; *** $P < 0.001$; **** $P < 0.0001$). These dose response experiments support the results shown in main Figure 3.

Supplementary Figure S7.

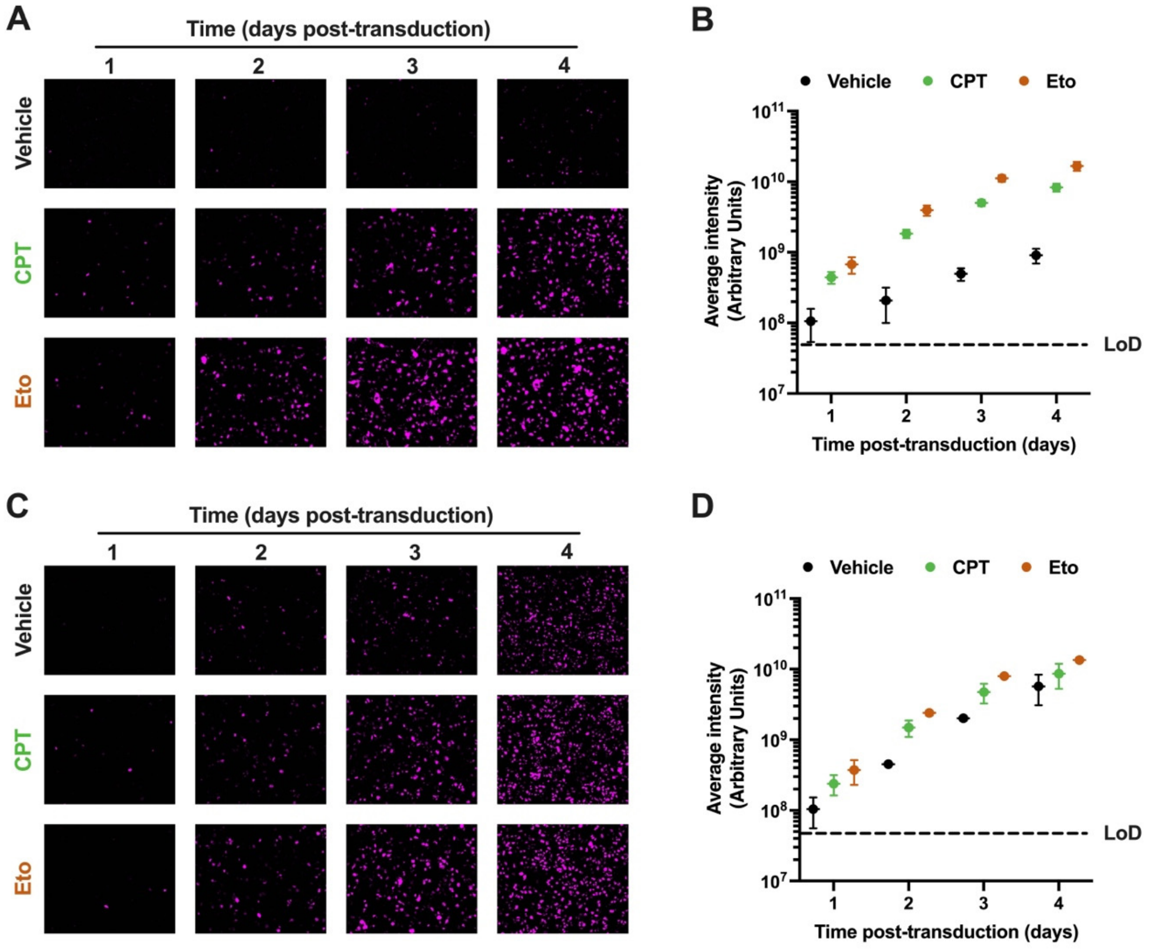

Figure S7. Analysis of CPT and Eto pre-treatments on IDLV and ICLV expression over time. Cells were treated for one hour with either vehicle, 2.5 µM of CPT or 50 µM of Eto and then transduced with IDLV-mCherry (25 µl virus/cell) **(A-B)** or ICLV-mCherry (3 µl virus/cell) **(C-D)**. mCherry accumulation was measured each day during the following four days. **(A and C)** Representative images of the mCherry accumulation (magenta) in **(A)** IDLV- and **(C)** ICLV-transduced cells are shown for each experimental condition at each time point. **(B and D)** Quantitation of the mCherry mean fluorescence intensity signal in each condition over the time frame of the experiment is shown. Data displayed in graphs are the mean values and standard deviation of at least three independent experiments performed in triplicates (n>9). Note that the effect of topoisomerase inhibitors on IDLV and ICLV is already detectable by day 1 post-transduction. These kinetic experiments support the results shown in main Figure 3.

Supplementary Figure S8.

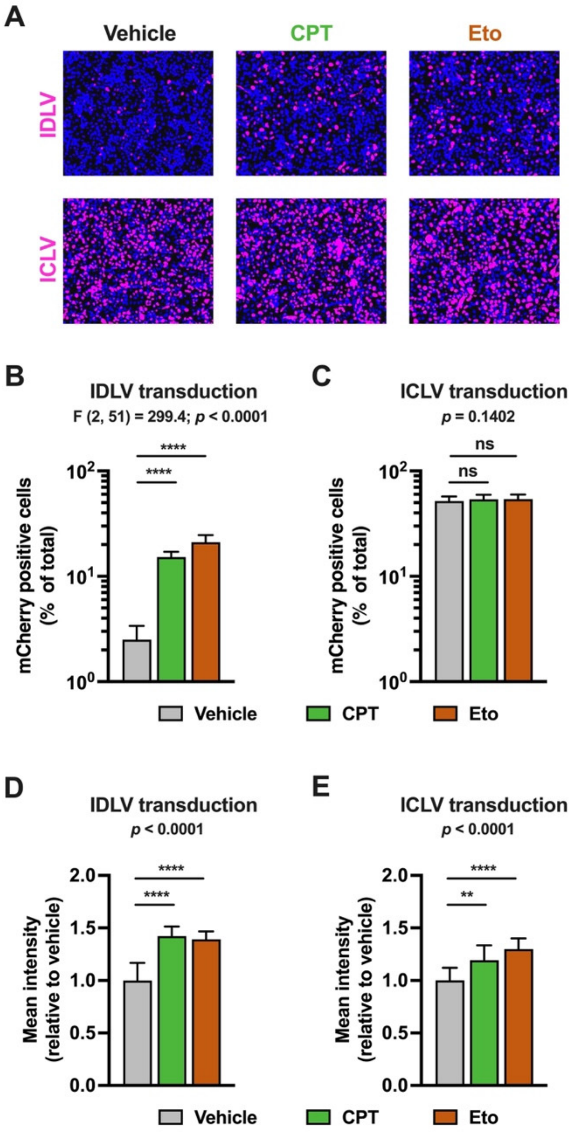

Figure S8. Analysis of CPT and Eto pre-treatments on IDLV and ICLV expression. Cells were treated for one hour with either vehicle, 2.5 µM of CPT or 50 µM of Eto and then transduced with ICLV-mCherry or IDLV-mCherry at the same 25 µl virus/well condition. mCherry accumulation was measured two days later. **(A)** Representative overlay images of mCherry accumulation (magenta) and nuclei staining (blue) in ICLV- (upper panels) and in IDLV- (lower panels) transduced cells. **(B and C)** Quantitation of the percentage of mCherry positive cells relative to the total number of cells in each experimental condition. **(D and E)** Quantitation of the mCherry mean fluorescence intensity signal in each condition relative to each corresponding vehicle-treated condition, that was set as 1. Data displayed in graphs are the mean values and standard deviation of three independent experiments with six replica per experiment (n=18). Data from B were normally distributed and were analyzed using one-way ANOVA followed by Dunnett's post-hoc test. Instead, data from C, D and E, that were not normally distributed, were analyzed using nonparametric Kruskal-Wallis test followed by Dunn´s multiple comparisons test. Statistical information is shown above each graph. Dunnett´s and Dunn´s tests were used to determine the statistical significance in pairwise comparisons (n.s.: not significant; ** $P < 0.01$; **** $P < 0.0001$). These experiments support the results shown in main Figure 4.
